# A Rare Presentation of Malignant Melanoma of the Face: A Case Report

**DOI:** 10.7759/cureus.64797

**Published:** 2024-07-18

**Authors:** Apurva Palatkar, Yash V Jain, Manu Babu, Vinod Shinde, Mayur Ingale

**Affiliations:** 1 Department of Otolaryngology, Head and Neck Surgery, Dr. D. Y. Patil Medical College and Hospital, Dr. D. Y. Patil Vidyapeeth, Pune, IND

**Keywords:** sunlight exposure, early diagnosis and treatment, head and neck tumors, cutaneous malignanat malenoma, rare casereport

## Abstract

Melanoma is a malignant neoplasm of melanoblasts, which are the precursors of the melanocytes arising from the neural crest cells. Melanomas can occur at various sites like the skin, eyes, upper esophagus, and meninges due to the migration of neural crest cells. Usually, the prognostic factors are decided based on the Breslow index. This case report describes a 61-year-old female who presented with the complaint of pinkish irregular swelling over the left side of her face for six months. The patient had a surgical resection, and the condition was determined to be invasive melanoma following confirmation by magnetic resonance imaging (MRI) and histological examination. Through our case report, we aim to shed light on the existing protocol for managing malignant melanoma while also exploring new aspects of presentation and multidisciplinary action.

## Introduction

Melanoma is a malignant neoplasm of melanoblasts, which are the precursors of the melanocytes arising from the neural crest cells [1]. The most common location of melanoma is the skin, but it can also be seen in the gastrointestinal tract, eyes, and brain due to the migration of neural crest cells during fetal life [2]. The process of transforming normal melanocytes into melanoma cells is still unexplained and is related to individuals with a fair complexion because they have very low melanin levels and are thus at increased risk of melanoma. The germline mutation of the CDKN2A gene is found in some families, and people with inherited disorders like xeroderma pigmentosa are at high risk for melanoma. This discussion is to enlighten the risk factors and clinical staging of melanomas, which eventually affect the treatment modalities and prognosis. The earlier detection of melanoma gives a better prognosis for the patient, as the five-year survival rate is 97% in stage 1 and 10-15% in stage 4 [1,3]. Melanoma is the least common, but it accounts for 75% of all skin cancer deaths [1]. Here, we present a case of a 61-year-old patient who presented with a pinkish irregular swelling over the left side of the nasal ala and vestibule and was meticulously managed by surgical resection and chemotherapy.

## Case presentation

A 61-year-old female came with the complaint of swelling over the left side of the face, which had been occluding the left nasal cavity for six months. It was initially small to begin with (peanut-sized) and gradually progressed reaching its current size of 3 x 3 cm. It was not associated with any pain or discharge from the swelling. She has a history of similar swelling in the same region two years ago; for which surgical excision was done. There was no history of trauma or epistaxis. There were no complaints of any other similar lesions on any part of the body. There was no history of a long-standing chronic illness or blood transfusion. Past and family history were not contributory.

**Figure 1:**
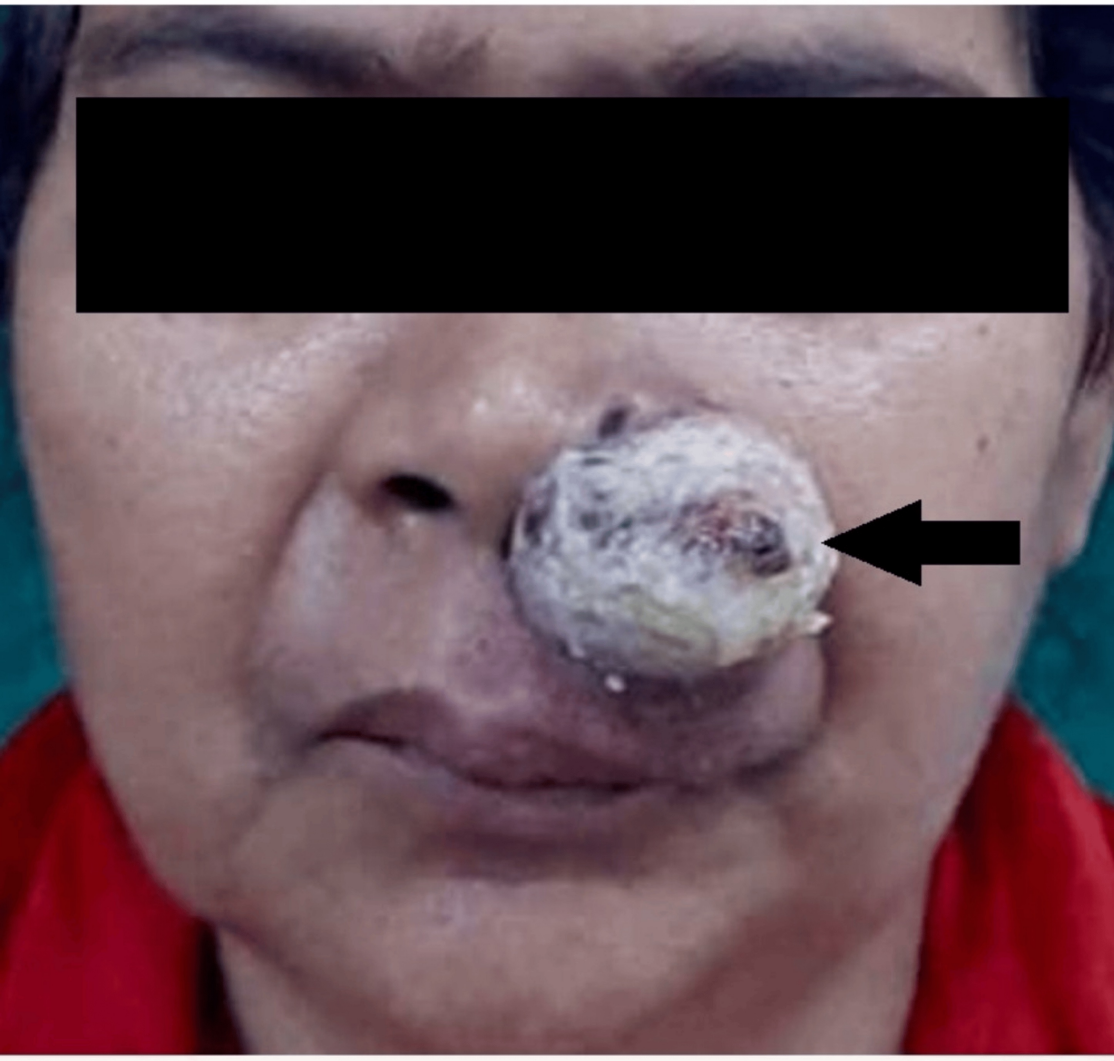
Preoperative image showing the lesion over the left ala of the nose The black arrow shows a lesion with a size of 3 x 3 cm approximately

On clinical examination, a 3 x 3 cm firm, pedunculated, pinkish, irregular globular swelling was seen arising from 0.5 cm inferior to the left nasal cavity over the left nasolabial fold. The left ala was pushed medially, occluding the left nasal cavity. Crusting was present over the surface of the swelling. The swelling was nontender, with no local rise in temperature and irregular margins (Figure 1). The posterior rhinoscopy was normal. The rest of the otorhinolaryngological, head and neck, and general examination were normal.

**Figure 2:**
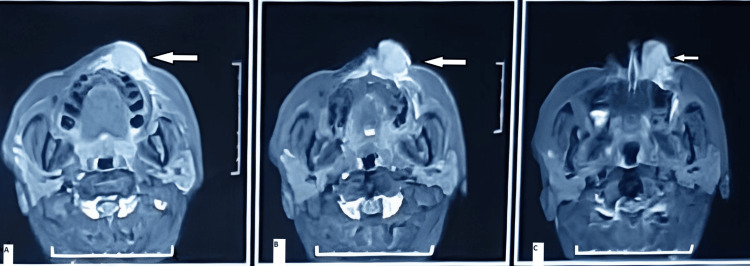
Axial cuts of the magnetic resonance imaging of the paranasal sinuses (A) A white solid fill arrow pointing toward the superior aspect of the lesion; (B) a white solid fill arrow showing altered signal intensity lesion in the left upper lip and upper lip adjacent infra-alar region; (C) a white solid fill arrow showing a lesion involving the floor of the left nose at the anterior aspect

All routine blood investigations were done and were within normal limits. Magnetic resonance imaging (MRI) of the paranasal nasal sinus (PNS) was done, which revealed a focal lobulated altered signal intensity lesion seen in the left upper lip adjacent to the infra-alar region involving the floor of the left nose at the anterior aspect. It shows heterogenous contrast enhancement. The adjacent bone appears to be normal. The lesion approximately measures 3.7 x 2.3 x 2.2 cm, representing neoplastic etiology (Figure 2).

**Figure 3:**
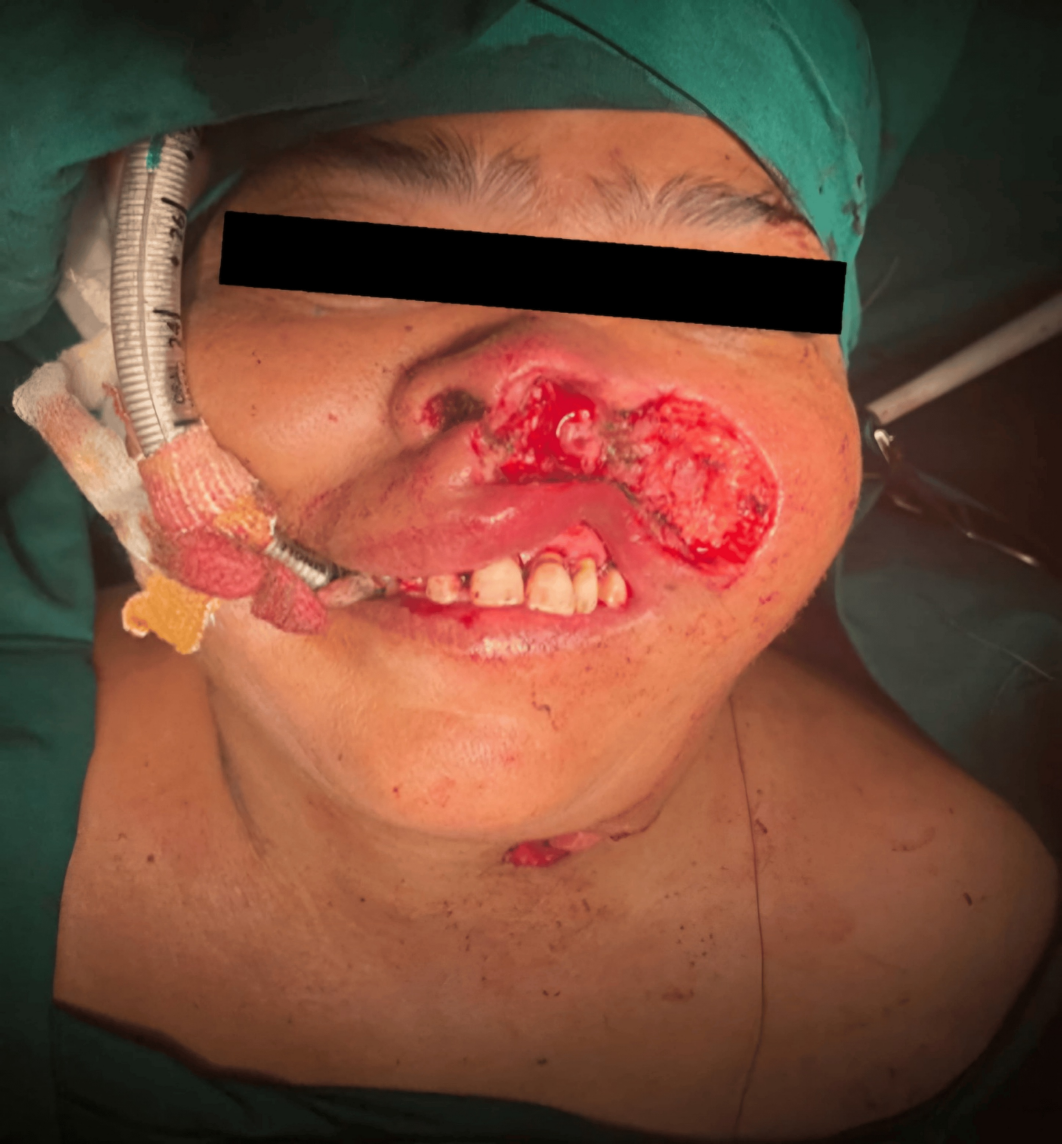
Intraoperative image showing the lesion excised from the left ala, left infra-alar region, and adjacent part of the left cheek

After obtaining consent from the patient, a biopsy of the mass was taken under local anesthesia, which was reported to be a neuroendocrine tumor. After obtaining consent from the patient and fitness for surgery from the anesthesiologist, the patient was taken up for surgery for complete resection and reconstruction (Figure 3). The mass, left ala, and adjacent part of the left cheek were sent for a frozen section, which came positive for invasive malignant melanoma, and then the aforementioned surgery was concluded with a left type II modified radical neck dissection.

**Figure 4:**
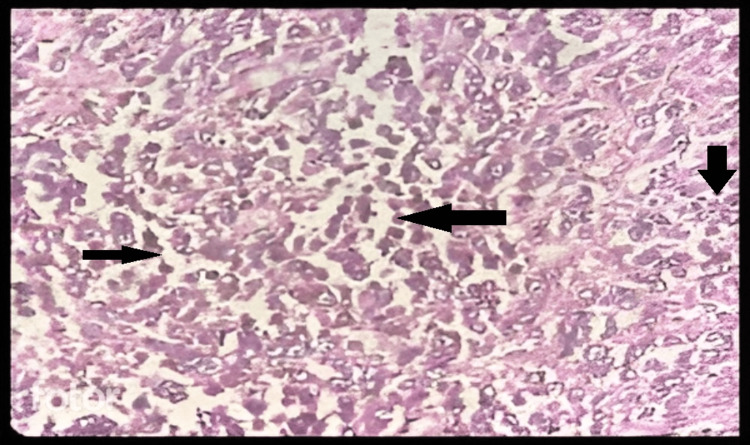
Histopathological image of the specimen The black arrows show brownish melanin pigment in the cells

**Figure 5:**
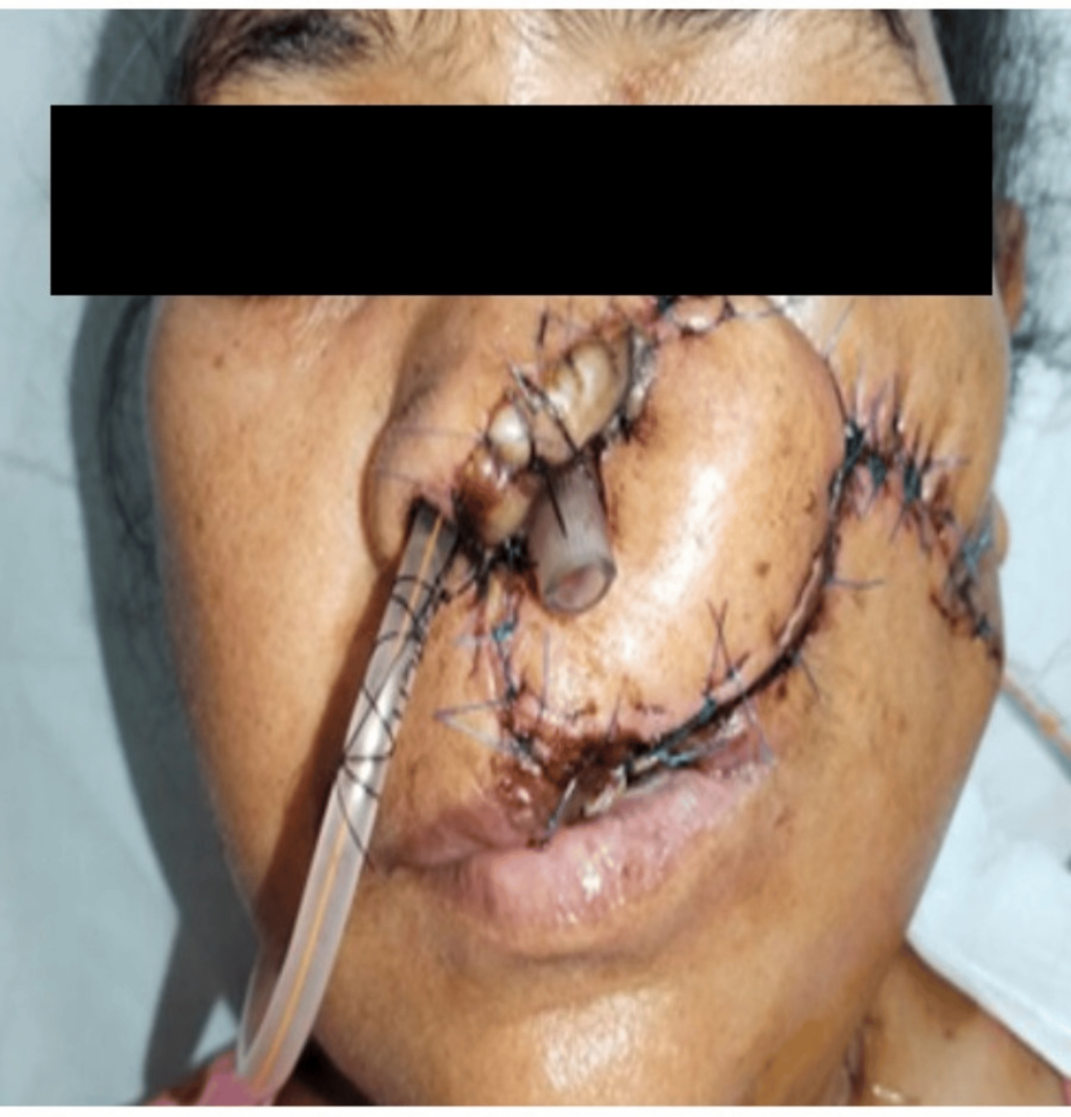
Postoperative image showing the reconstruction after the excision of the lesion

The histopathology of the lesion showed an invasive neoplasm with tumor cells arranged in lobules and nests and covered by stratified squamous epithelium at a few foci. The tumor cells are round to oval at places, spindle-shaped, highly pleomorphic, and have large vesicular nuclei. At a few foci, the new nuclei appear round and hyperchromatic with anaplastic features. Macro nucleoli are seen in a few places. A few sections show pigment deposition in some cells. Stroma shows mild lymphocytic infiltration. Perineural infiltration is noted at a few foci. Two lymph nodes were positive (Figure 4). On immunohistochemistry findings, S-100 and human melanoma black (HMB) 45 were positive, thus suggesting invasive malignant melanoma pT4bpN2a. Postoperatively, the patient had no complications (Figure 5).

## Discussion

Cutaneous melanoma, a tumor arising from melanocytes, is one of the most aggressive tumors seen in humans. The melanocytes originate from the neural crest located along the mucosal surface, layer of the eye, and basal epidermis [4]. Although cutaneous melanoma can occur anywhere on the skin surface, the specific location on the body is found to be affected by the age and sex of the patient [5]. It can occur in any age group, but the median age at diagnosis is 59 years [1,6].

According to the Tumor, Node, and Metastasis (TNM) classification, there are four stages in which the primary tumor is staged; based on Breslow thickness and the presence or absence of ulceration, the staging goes from Tx to T4 [1]. There are a few types of melanomas, including amelanotic, desmoplastic, acral, lentiginous, nodular, lentigo maligna, and superficial spreading. Approximately 70% of melanomas are superficial spreading types [7].

Once the melanoma spreads or metastasizes from the origin, the response rate to treatment is decreased by approximately 5-20%, and the 10-year survival rate goes around 10%. To lower the morbidity and mortality rate in melanoma, it is important to detect the lesion early when the neoplastic cells are limited to the epidermis [8].

If the lesion is suspected of clinical melanoma, a skin biopsy, a complete excision biopsy with negative margins, is preferred, though different molecular and imaging modalities are known [9-11]. Excision of the lesion is the treatment of choice and is the ultimate remedy for patients who are newly diagnosed and at an early stage of malignancy [12].

The National Institute for Health and Care Excellence (NICE) recommends at least 5 mm margins for stage 0 melanomas, 10 mm for stage 1, and 20 mm for stage 2. Other than surgical excision, immunotherapy and kinase therapy have a positive response, while chemotherapy is considered a second-line treatment [13]. After the oncological decision for stage 4 patients, palliative or radiotherapy is considered as dacarbazine, a chemotherapeutic agent option, has limited benefits. 

In such cases, new targeted therapies have emerged due to the discovery of new signaling pathways and mutations in those pathways. These agents are serine/threonine-protein kinase B-Raf (BRAF) and mitogen-activated protein kinase (MEK) inhibitors and immune checkpoint inhibitors such as cytotoxic T lymphocyte-associated antigen 4 antibodies (CTLA4) and programmed cell death ligand 1 (PD1) antibodies. PD1 and CTLA4 antibodies such as pembrolizumab, ipilimumab, and nivolumab, along with specific BRAF inhibitors like dabrafenib and vemurafenib alone and/or blended with MEK inhibitors such as cobimetinib and trametinib, have a promising outcome [1,14-20].

## Conclusions

In conclusion, cutaneous malignant melanoma poses a significant challenge despite advancements in detection and treatment. We have identified several crucial factors influencing its incidence, progression, and prognosis. Our research highlights the latest therapeutic innovations and novel therapies such as immunotherapy which shows promise in improving outcomes for patients with advanced disease. Continued research into molecular pathways and biomarkers is crucial for personalized treatment strategies. Multidisciplinary collaboration is essential to enhance early detection and treatment efficacy. The diagnosis and management protocol of malignant melanoma has proven to be a challenge in the past years. With the help of collaborative efforts from various departments, we can strive toward an early diagnosis, safe and effective personalized treatment plans, and ultimately improved quality of life of patients affected by mucocutaneous malignant melanoma.
